# Heterogeneous Expression of Proangiogenic and Coagulation Proteins in Gliomas of Different Histopathological Grade

**DOI:** 10.3389/pore.2021.605017

**Published:** 2021-03-31

**Authors:** Marek Z. Wojtukiewicz, Marta Mysliwiec, Elwira Matuszewska, Stanislaw Sulkowski, Lech Zimnoch, Barbara Politynska, Anna M. Wojtukiewicz, Stephanie C. Tucker, Kenneth V. Honn

**Affiliations:** ^1^ Department of Oncology, Medical University of Bialystok, Bialystok, Poland; ^2^ Department of Clinical Oncology, Comprehensive Cancer Center of Bialystok, Bialystok, Poland; ^3^ Department of General Pathomorphology, Medical University of Bialystok, Bialystok, Poland; ^4^ Department of Philosophy and Human Psychology, Medical University of Bialystok, Bialystok, Poland; ^5^ Robinson College, University of Cambridge, Cambridge, United Kingdom; ^6^ Department of Pathology-School of Medicine, Bioactive Lipids Research Program, Wayne State University, Detroit, MI, United States; ^7^ Karmanos Cancer Institute, Detroit, MI, United States; ^8^ Department of Chemistry, Wayne State University, Detroit, MI, United States

**Keywords:** angiogenesis, VEGF, bFGF, D-dimers, blood coagulation, fibrin, tissue factor, glial tumors 3

## Abstract

Brain gliomas are characterized by remarkably intense invasive growth and the ability to create new blood vessels. Angiogenesis is a key process in the progression of these tumors. Coagulation and fibrinolysis factors play a role in promoting angiogenesis. The aim of the study was to evaluate the expression of proangiogenic proteins (VEGF and bFGF) and hemostatic proteins (TF, fibrinogen, fibrin, D-dimers) associated with neoplastic cells and vascular endothelial cells in brain gliomas of various degrees of malignancy. Immunohistochemical tests were performed using the ABC method with the use of mono- and polyclonal antibodies. The obtained results indicated that both neoplastic cells and vascular endothelial cells in gliomas of various degrees of malignancy are characterized by heterogeneous expression of proteins of the hemostatic system and angiogenesis markers. The strongest expression of proangiogenic factors and procoagulant factors was demonstrated in gliomas of higher-grade malignancy.

## Introduction

In view of the fact that the hemostatic system is anatomically and functionally related to vascularization, its influence on the process of angiogenesis in neoplastic tumors would appear interesting. Since 1971, when J. Folkman promulgated the hypothesis that the growth of solid tumors depends on angiogenesis, and that therapeutic measures aimed at inhibiting this process may be an effective element of anticancer therapy [1, 2], the era of intensive research into anti-angiogenic treatment was initiated. From the outset, this therapy is considered appropriate for glial tumors. Among them, greatest interest has been paid to immature polymorphic glioblastomas (*glioblastoma multiforme*), which constitute more than half of all gliomas, show the highest degree of malignancy and have exceptionally poor prognosis.

Glial tumors are characterized by remarkably intense invasive growth and the ability to create new blood vessels. In glioblastoma multiforme, vascular density is decidedly higher than in tumors with lower histological malignancy [3]. Studies on astrocytomas have described an inverse correlation between vessel density and overall survival time and indicate that the number of vessels in the tumor biopsy may be an important prognostic factor [4, 5]. Angiogenesis is a key process in the progression of malignant neoplasms, namely an essential stage for tumor growth above 2–3 mm^3^ and the development of metastases [6]. Activation of this process may result not only from an increase in the activity of factors stimulating angiogenesis, but also from a decrease in the activity of inhibitors of this process [7].

Among the proangiogenic factors, the main contributing mediators in vascularization of the neoplastic tumor are the growth factors: vascular endothelial growth factor (VEGF) and basic fibroblast growth factor (bFGF). Studies on glioblastoma multiforme have demonstrated that VEGF production is particularly induced in tumor cells close to the foci of necrosis. The coexistence of glomerular proliferation with areas of extensive necrosis indicates that the angiogenic response is a secondary effect of increased VEGF production by hypoxic tumor tissue [8]. It is known that hypoxia plays an important role in the expression of the VEGF gene, inducing its activity at the level of transcription with the participation of HIF-1 (hypoxia inducible factor-1) [9]. VEGF increases the permeability of blood vessels (it is approx.50,000 times more potent as a factor affecting small blood vessel permeability than histamine) [10].

An increase in VEGF expression under the influence of bFGF has been demonstrated, as well as a synergistic effect of both factors in the angiogenesis process [11]. Despite the fact that bFGF also stimulates the proliferation of cells other than endothelial cells, it meets the basic criteria characterizing the proangiogenic factor: it stimulates the proliferation of endothelial cells *in vitro*, induces angiogenesis *in vivo*, and is often present in areas of vascular growth [8, 12]. bFGF is implicated in brain tumor progression and localizes in the microvasculature as well as in the tumor cells in human gliomas [8]. FGF receptor (FGFR) plays an important role in the survival and angiogenesis of glioblastoma cells through phosphatidylinositol 3-kinase (PI3K)/protein kinase B or AKT/mammalian target of the rapamycin (mTOR) molecular signaling pathway [13, 14].

Tissue Factor (TF) expression has been demonstrated on the surface of many normal cells as well as neoplastic cells [15]. The process of developing malignant brain tumors damages normal brain tissue and the structure of its blood vessels. Therefore, a growing tumor may lead to an increase in TF expression [16]. The protumorigenic function of TF is to activate cell signaling through the interaction of its cytoplasmic domain with protease-activated receptors (PARs). Antibodies blocking the cytoplasmatic signaling domain of TF have been shown to result in reduced tumor growth, but had no effect on the ability of TF to initiate coagulation [17]. Tissue factor is assigned a special role in the angiogenesis process. It has been shown to participate in the processes of adhesion and migration of neoplastic cells through a mechanism independent of clotting activation [18]. At the same time, it has been demonstrated that over-expression of the gene encoding TF leads to increased transcription of the gene encoding the VEGF factor, as well as to the reduction of the gene responsible for the synthesis of the angiogenesis inhibitor—thrombospondin-1 [15].

The procoagulant activity of thrombin leads to the conversion of fibrinogen into fibrin. Increased fibrinogen turnover and shortened plasma half-life are often found in cancer patients [19]. The presence of fibrin in the extravascular space, confirming that the activation of blood coagulation has taken place there, has been documented in a number of malignant neoplasms [20–24]. Fibrin and fibrinogen induce the expression of TF, which consequently leads to overproduction of fibrin. The resulting fibrin network provides “scaffolding” that promotes the growth of new blood vessels. Additionally, fibrin and fibrinogen facilitate the activation and stability of bFGF and other proangiogenic factors.

The aim of the study was to evaluate the expression of proangiogenic proteins (VEGF and bFGF) and coagulation/fibrinolysis proteins (TF, fibrinogen, fibrin, D-dimers) associated with neoplastic cells and vascular endothelial cells in brain gliomas of various degrees of malignancy.

## Materials and Methods

Glioma tissues and tissues from the margin of these tumors were obtained at surgical resection of 40 cancer patients. The material consisted of 13 lower-grade and 27 higher-grade malignant tumors.

Immunohistochemical (IHC) studies were performed on G2-grade gliomas (8 astrocytomas, 5 oligodendrogliomas) and high-grade gliomas (G3–12 anaplastic astrocytomas, 4 anaplastic oligodendrogliomas; G4–11 glioblastomas multiforme), as well as control fragments of respective normal tissues, which were derived from the neoplasm-free surgical margins.

The study protocol was approved by the local Ethics Committee of the Medical University in Bialystok, Poland (approval number R-I-002/256/2003). Informed consent was obtained from the patients.

Antigens were detected with avidin-biotin complex technique (ABC) using reagents (Vectastain Kits, Vector Laboratories, Burlingame, CA, United States) which have been described previously [25].

The following mouse monoclonal antibodies were used:

• 1-8C6 antibody to fibrinogen and fibrin-I, which requires intact 14Arg-15Gly binding on the Bβ chain of fibrinogen and therefore does not react with fibrin II (lacking fibrinopeptide B, FPB).
• T2G1 antibody to fibrin-II, which reacts with the amino-terminal part of the Bβ chain of fibrinogen only after cleavage by thrombin of fibrinopeptide B (FPB, Bβ 1–14) and therefore does not react with fibrinogen and fibrin I.
• GC4 antibody to D-dimers
• Antibody to human recombinant TF – kindly provided by Dr. Walter Kisiel, University of New Mexico, Dept. of Pathology, School of Medicine, Albuquerque, United States. The antibody was employed in our earlier studies [26].

Antibodies (1-8C6, T2G1, GC4) were kindly provided by Dr. Bohdan Kudryk, Lindsley F. Kimball Research, Blood Center, NY, United States and used in our earlier studies [22].

The following polyclonal antibodies were used:

• Goat antibody to human recombinant VEGF 121 and VEGF 165–R&D Systems, United States
• Goat antibody to human recombinant bFGF-R&D Systems, United States

The results of staining of the glioma tissues were compared with matched normal tissues, which were processed simultaneously. Antigens of the proteins tested were detected as the brown reaction product of the avidin-biotin complex with the substrate. Visual assessment of protein expression was performed in 10 random high-power fields by two independent observers.

Intensity of IHC reactions was evaluated acc. to Hirsch et al [27] in modification by Pirker et al [28]. A score for each tissue core was generated using a semi-quantitative approach according to the following algorithm: the percentage of positive tumor cells per slide (0–100%) was multiplied by the dominant intensity pattern of staining (0–negative for trace; 1-weak; 2–moderate; 3 intense). Hence the range for the overall score was 0–300. Specimens with a score of 0–199 were classified as being negative, while those with a score between 200–300 as positive. The IHC score was calculated based on the following formula: 1x (percentage of cells staining weakly [1+]) +2 x (percentage of cells staining moderately [2+] + 3 x (percentage of cells staining strongly [3+]) [28].

χ2 test was employed for statistical analysis. *p* value of <0.05 was considered statistically significant.

## Results

The strongest expression of the VEGF antigen was obtained in glioblastoma multiforme cells and the endothelium of their blood vessels, with weaker but distinct expression in G3 tumors (anaplastic gliomas) and the weakest in endothelial cells and a few G2 glioma neoplastic cells (Table 1).

**TABLE 1:** Number of tumors exhibiting distinct intensity of IHC reactions toward proteins of hemostatic system and angiogenesis markers in gliomas of different malignancy.

Proangiogenic factors	Localization	Low-grade gliomas (n = 13) IHC score		High-grade gliomas (n = 27) IHC score		*p* value
		<200	≥200	<200	≥200	
VEGF	Cancer cells	9 (69, 2%)	4 (30, 8%)	2 (7, 4%)	25 (92, 6%)	*p* <0,001
	Endothelial cells	2 (15, 4%)	11 (84, 6%)	1 (3, 7%)	26 (96, 3%)	NS
	Neuropil	13 (100%)	0	1 (3, 7%)	26 (96, 3%)	*p* <0,001
bFGF	Cancer cells	2 (15, 4%)	11 (84, 6%)	24 (88, 9%)	3 (11, 1%)	*p* <0,001
	Endothelial cells	1 (7, 7%)	12 (92, 3%)	25 (92, 6%)	2 (7, 4%)	*p* <0,001
	Neuropil	13 (100%)	0	27 (100%)	0	NS
TF	Cancer cells	8 (61, 5%)	5 (38, 5%)	3 (11, 1%)	24 (88, 9%)	*p* = 0,03
	Endothelial cells	13 (100%)	0	5 (18, 5%)	22 (81, 5%)	*p* <0,001
	Neuropil	2 (15, 4%)	11 (84, 6%)	4 (14, 8%)	23 (85, 2%)	NS
Fibrinogen	Cancer cells	12 (92, 3%)	1 (7, 7%)	22 (81, 5%)	5 (18, 5%)	NS
	Tumor stroma in the vicinity of blood vessels	10 (76, 9%)	3 (23, 1%)	23 (85, 2%)	4 (14, 8%)	NS
Fibrin	Cancer cells	11 (84, 6%)	2 (15, 4%)	24 (88, 9%)	3 (11, 1%)	NS
	Tumor stroma in the vicinity of blood vessels	4 (30, 8%)	9 (69, 2%)	6 (22, 2%)	21 (77, 8%)	NS
D-dimers	Cancer cells	3 (23, 1%)	10 (76, 9%)	2 (7, 4%)	25 (92, 6%)	NS
	Tumor stroma in the vicinity of blood vessels	5 (38, 5%)	8 (61, 5%)	3 (11, 1%)	24 (88, 9%)	NS

Clear and consistent expression of bFGF was found in tumor cells and in lower-grade malignant glioma vascular endothelial cells, while in gliomas of higher-grade, bFGF expression was less pronounced (Table 1).

**FIGURE 1:**
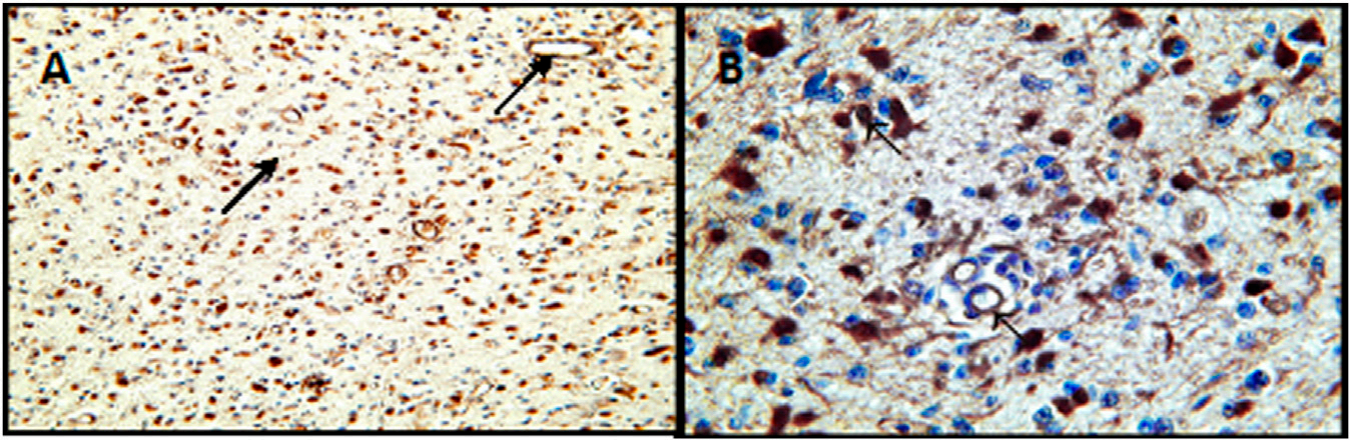
Expression of tissue factor in G3 astrocytomas. **(A)** Positive immunohistochemical reaction for the presence of TF in neoplastic cells and vasuclar endothelial cells-arrows at lower magnification (×100). **(B)** Positive innunohistochemical staining for the presence of TF at higher magnification (×400). Arrows show staining of staining of tumor cells and vascular endothelial cells.

A positive color reaction to the presence of TF antigen was demonstrated in neoplastic cells and vascular endothelial cells in high-grade glioma tissues (Figures 1A,B; Table 1) and a non-uniform, diffuse positive reaction in neuropil and in the necrotic areas of these high-grade gliomas.

A clear, positive reaction to fibrinogen antigens was above all demonstrated in the lumen of the blood vessels of all the examined tumors (Table 1), as well as in areas of vascular effusions. The presence of fibrinogen in the extravascular space was confirmed in the form of a weak positive reaction in the vicinity of small blood vessels and in the neuropil of anaplastic gliomas. There was no correlation between the presence of fibrinogen antigens and the intensity of the color reaction in relation to the degree of malignancy of the gliomas.

Fibrin antigens were located in the neuropil around small blood vessels, in necrotic foci and in individual tumor cells (Table 1). No systematic relationship was observed with regard to the degree of malignancy of the gliomas, either in the distribution of the antigens or in the degree of intensity of the color reaction.

The strongest expression of D-dimer antigens was found in higher-grade gliomas (Table 1).

## Discussion

The importance of angiogenesis as an independent prognostic factor in brain gliomas has long been recognized. An inverse correlation has been found between the intensity of angiogenesis and the survival time of patients with gliomas [8]. Different factors involved in the hemostatic system, as well as the impact of individual elements of this system on cancer cells and the vascular endothelium are extremely important in the process of angiogenesis. The contribution of these factors and their reaction products in relation to tumor growth, metastasis and new vessel formation in tumor tissue has been documented [8, 19, 29, 30]. These angiogenic factors are upregulated by a variety of mechanisms like oncogene activation, loss of tumor suppressor gene function, and/or a hypoxic microenvironment [31]. It has been shown that the expression of growth factors in glioblastoma tissues increases with the growth of their malignancy, and the expression of some hemostatic system factors correlates with the expression of growth factors [8].

On the basis of our own research and data from the literature [32, 33], it has been confirmed that VEGF expression in glioma cells correlates with the degree of tumor malignancy. The strongest expression of VEGF was observed in the cells of glioblastoma multiforme and the endothelial cells of their blood vessels, with weaker but distinct expression in anaplastic gliomas and the weakest in a few neoplastic cells and vascular endothelial cells of lower-grade malignant gliomas. Similar results have been reported by Carrol et al. [34]. Studies conducted on various glioblastoma cell lines and in human glioblastoma tissues revealed the interaction of VEGF with angiopoietins: Ang-1 and Ang-2 and their Tie-2 receptor, with the consequent effect of aiding the maturation and stabilization of blood vessels [35]. Ang-2 is an antagonist of the Tie-2 receptor. As the tumor grows, the amount of synthesized Ang-2 increases, which leads to destabilization of the capillary wall. As a result of this process, in the absence of VEGF, the blood vessels regress and form necrotic foci in the center of the tumor. However, in the presence of VEGF, which is expressed later than Ang-2, an increase in Ang-2 secretion leads to increased angiogenesis, especially at the periphery of the tumor [35, 36]. Increased VEGF expression has been associated with aggressiveness of the tumor and poorer prognosis in patients with uterine cancer, ovarian cancer [37], breast cancer [37–39], gastric cancer [40], melanoma [41], head and neck cancer [37], and non-small cell lung cancer [38]. In addition, a high level of VEGF coexists with shortened survival time and an increased likelihood of recurrence of malignant tumors of the colon, rectum and kidney [37], and may also contribute to the initiation of the metastatic process [42].

One of the most exciting developments is the discovery, that autocrine and paracrine VEGF signaling contributes to vital aspects of tumorigenesis, namely cancer stem cells (CSCs) function, independently of angiogenesis. An alternative form of blood supply is vasculogenic mimicry, a process resembling embryonic vascular network, which is carried out by CSCs [43]. The cells are capable to transdifferentiate and form vascular-tube structures in the absence of endothelial cells [43]. It is of interest, that glioblastoma stem cells (GSCs) are also endowed with an ability to differentiate into endothelial cells and thus promote angiogenesis [43].

The neuropilins (NRPs), in addition to VEGF receptor tyrosine kinases, are fundamental for mediating the effects of VEGF on CSCs, due to their ability to affect the function of growth factor receptors and integrins [45]. VEGF signaling mediated by the NRPs impacts tumor cells, independently of its function in angiogenesis and vascular permeability. It is noteworthy that VEGF–NRP2 signaling in tumor cells is associated with poor prognosis and therapy resistance. In such case, targeting VEGF may turn out ineffective [46].

The expression of bFGF in the conducted studies was demonstrated in glioma cells of various degrees of malignancy, as well as in vascular endothelial cells. There was no correlation between bFGF and VEGF expression in glioma tissues. Other authors have also failed to demonstrate a correlation between bFGF expression and the intensity of angiogenesis in gliomas [47]. Additionally, similar results have been obtained for rectal cancer [48] and gastric cancer [49]. In turn, Ahir et al. [8] have shown in their studies that the combination of VEGF with bFGF with/or without platelet-derived growth factor demonstrated a synergistic effect in inducing neovascularization *in vivo*. The expression of these growth factors correlates with tumor progression in high-grade tumors expressing higher levels of growth factors when compared to low-grade tumors. Yet other studies have revealed that bFGF is implicated in brain tumor progression and is localized in the microvasculature as well as in tumor cells in human gliomas [50–52]. bFGF levels correlate with the degree of glioma malignancy and vascularity as determined by immunohistochemical analysis [51]. It has previously been demonstrated that antibodies for bFGF inhibit glioma growth *in vivo* model and lead to reduced blood vessel densities in glioma tumors of treated animals [53].

In our own studies, the presence of TF antigens was demonstrated in neoplastic cells and vascular endothelial cells of all higher-grade malignant gliomas, but only in a few lower-grade malignant glioma neoplastic cells. TF antigens were also demonstrated in neuropil, and the intensity of the color reaction was directly correlated with the tumor grade, which is consistent with studies by Hamada et al. 1996 [54]. Similar results were obtained by other researchers who demonstrated strong or moderate TF expression in 91% of G4 gliomas, 46% of G3 gliomas, and only 16% of G1 and G2 gliomas [55]. Studies have shown that brain gliomas are a rich source of TF [54, 56] and the level of TF is directly correlated with the grade of their histological malignancy [56, 57].

In our own research, we have also demonstrated a correlation between the expression of TF and VEGF antigens, which is indicative of a relationship between TF and the intensity of angiogenesis in gliomas. The results obtained by other researchers have also confirmed this relationship [57, 58]. In a group of 23 glioblastoma multiforme tumors, the vascular density of tumors with strong TF-expression in endothelial cells was significantly higher than in those whose endothelial cells did not express TF [57]. The same study confirmed the correlation between the presence of TF and VEGF in glioblastoma extracts, which indicates the likely cooperation of both factors in the angiogenesis process in brain gliomas.

TF is believed to indirectly influence tumor angiogenesis through its procoagulant activity leading to thrombin generation and the formation of proangiogenic fibrin [59–61]. The results of the current study confirm the presence of fibrin and fibrinogen antigens in the extravascular space of the examined glioma tissues. However, no correlation was found between the intensity of expression of these antigens and the degree of malignancy of the gliomas. Other researchers have demonstrated the presence of stabilized fibrin deposits in glioblastoma multiforme between the tumor foci as well as on the periphery of the tumors [62]. In the same study, however, there was a different distribution of fibrin in brain metastatic tumors whose primary site was the lung. Fibrin deposits were only observed around the periphery of these tumors. Fibrin deposits around the tumor and in the tumor stroma provide double protection for the tumor against the host’s immune system, namely they create a barrier against cells of the immune system and mask tumor antigens, and support angiogenesis [19, 63].

Thrombin generation, formation of a fibrin network and secondary fibrinolysis are evidenced by the presence of fibrin breakdown products found around small blood vessels and in association with glioblastoma cancer cells. Expression of D-dimers was clearly stronger in higher-grade gliomas. Compared to lower-grade malignant gliomas, anaplastic and multiforme gliomas are characterized by a stronger expression of proangiogenic factors and procoagulant factors involved in the processes of tumor growth and progression. When analyzing the expression of D-dimers and fibrin in the tissues, an inverse correlation was observed between these antigens: low fibrin expression was accompanied by high D-dimer expression, while strong fibrin expression was accompanied by low D-dimer expression.

The above results indicate that neoplastic cells and vascular endothelial cells in gliomas of various degrees of malignancy are characterized by heterogeneous expression of proteins of the hemostatic system and angiogenesis markers. Compared to lower-grade gliomas, higher-grade gliomas express more proangiogenic factors and procoagulant factors involved in tumor angiogenesis and progression. In anaplastic oligodendrogliomas, the expression and distribution of the tested antigens is similar to that in low-grade gliomas.

There are multiple links between coagulation activation and angiogenesis in cancer. Coagulation activation with subsequent fibrin formation takes place in the extravascular compartment of glial tumors. This solid phase coagulopathy may, at least in part, account for more pronounced angiogenesis observed in higher-grade gliomas, and thus contribute to more malignant course of the tumors.

## Data Availability

The original contributions presented in the study are included in the article/Supplementary Material, further inquiries can be directed to the corresponding author.
